# The chloroplast genome of the allotriploid, *Paeonia × lemoinei* cv. Oukan, and its phylogenetic implications

**DOI:** 10.1080/23802359.2025.2519229

**Published:** 2025-06-23

**Authors:** Mingyang Hao, Jiang Wu, Mengyao Wang, Ying Chen, Qi Qiao, Sophie Newmarch, Erqiang Wang, Richard Winkworth, Bingyou Fan

**Affiliations:** a College of Agriculture, Henan University of Science and Technology, Luoyang, China; b School of Information Engineering, Yulin University, Yulin, China; c School of Agriculture and Environment, Massey University, Palmerston North, New Zealand; d Peony Gene Development Engineering and Technology Center of Henan Province, Central Plains Scholar Workstation, Luoyang Academy of Agriculture and Forestry Sciences, Luoyang, China

**Keywords:** *Paeonia × lemoinei*, cv. Oukan, triploid, chloroplast genome, phylogenetic relationship

## Abstract

The chloroplast genome of *Paeonia* × *lemoinei* cv. Oukan, a newly identified allotriploid from *Paeonia* sect. *Moutan*, shows typical tree peony features in size, structure, and gene content. A maximum-likelihood phylogenetic analysis revealed cv. Oukan and the known triploid cv. Shouanhong clustered into subsect. *Delavayanae* and subsect. *Vaginatae*, respectively. Cv. Oukan is closely related to its maternal parent cv. High Noon, while autotriploid cv. Shouanhong is sister to *P. ostii* but genetically distinct. This study provides genomic resources and phylogenetic insights for the first allotriploid tree peony, suggesting lineages in both subsects could serve as maternal parents for breeding triploid cultivars.

## Introduction

*Paeonia suffruticosa* Andrews (1804), namely tree peony, is native to China and has been cultivated worldwide with considerable value as an ornamental as well as in the medicinal and edible oil industries (Chen et al. 2023). With a pair of exceptions, the species and cultivars of tree peony are diploid. Perhaps the most well-known exception is the autotriploid *P. suffruticosa* cv. Shouanhong, which has high ornamental value (Li and Zhang 1982). Recently, *Paeonia × lemoinei* Rehder (1820) cv. Oukan, belonging to cultivated tree peony varieties, with *P. × lemoinei* cv. High Noon as maternal parent and *P. suffruticosa* cv. Shinfuso as paternal parent, was identified, based on GISH (genomic *in situ* hybridization) and FISH (fluorescence *in situ* hybridization), as the second triploid and first allotriploid in sect. *Moutan* (Zhong et al. 2024). This allotriploid has larger flowers, taller and more robust flowering shoots, and grows more vigorously than either its maternal parent, or its paternal parent (Zhong et al. 2024). Polyploid organisms often exhibit heterosis, outperforming their diploid relatives in various aspects (Sattler et al. 2016) and polyploid breeding is an important approach to trait improvement in cultivated plants (D’Agostino and Fasano 2024). However, to date, there has been no feasible method for generating polyploid tree peonies (Yang et al. 2020).

Chloroplasts are the cytoplasmic organelles in plant and algal cells within which photosynthesis occurs (Allen et al. 2011). Chloroplast genome sequences have long been important for studies of plant phylogeny and evolution (Daniell et al. 2021). Although, complete chloroplast genome sequences are available for wild tree peony species and several cultivars (Cai et al. 2023; Chen et al. 2023), the chloroplast genome of the allotriploid *Paeonia × lemoinei* cv. Oukan has yet to be reported. In this study, we sequenced, assembled, and analyzed the chloroplast genome of cv. Oukan and investigated molecular phylogenetic relationships among the two triploid cultivars and wild tree peony species.

## Materials and methods

### Plant material, DNA extraction, and sequencing

**Figure 1.**
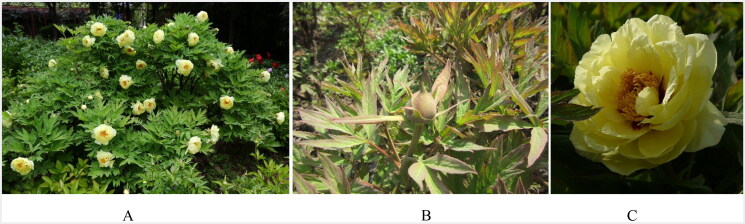
*Paeonia × lemoinei* cv. Oukan. (A) Growth habit. Tall, upright growing plant to 150 cm tall and 90 cm wide at maturity. Mid to late season blooming, similar to Japanese cultivars. Flowers supported on strong stems that do not droop as in many *Lutea* hybrids. (B) The leaves are large with nine leaflets, each deeply and sharply lobed, dark green with margin rolled inward. (C) Lemon yellow, semi-double blossoms up to 20 cm cross with a slight red flare at the base of the petals. Slight lemon fragrance similar to the maternal parent, cv. High Noon although the flowers are larger and paler in color than its maternal parent. Photos taken by Erqiang Wang at the Luoyang Academy of Agricultural and Forestry Sciences (N34°22′48″, E112°17′24″).

Fresh leaves of cv. Oukan were collected from the Tree Peony Germplasm Nursery at the Luoyang Academy of Agricultural and Forestry Sciences (N34°22′48″, E112°17′24″) (Figure 1). A specimen was deposited at the Herbarium of Luoyang Key Laboratory of Peony Biology, Henan University of Science and Technology (http://www.haust.edu.cn, Bingyou Fan, bingyou.fan@haust.edu.cn) under the voucher number LKLPB202403. Total DNA was extracted from fresh leaves using the modified CTAB method (Doyle and Doyle 1987) with agarose electrophoresis used to check DNA integrity and DNA concentration was estimated using a Nanodrop™ ND2000C spectrophotometer (ThermoFisher Scientific, Waltham, MA). Total DNA was sequenced by Bio&Data Biotechnologies (Guangzhou, China) using a BGISEQ-500 sequencer and following manufacturer’s recommendations for library preparation, hybridization, and sequencing. The raw data were filtered to remove adapter sequences and low-quality reads using SOAPnuke v.2.0 (Chen et al. 2018).

### Genome assembly and annotation

A circular chloroplast genome sequence for cv. Oukan was assembled with GetOrganelle (Jin et al. 2020) using default parameters. A genome annotation for the cv. Oukan genome sequence was prepared using GeSeq (Tillich et al. 2017) and tRNAscan-SE (Lowe and Eddy 1997).

### Phylogenetic analysis

Chloroplast genome sequence for 10 tree peonies of sect. *Moutan* plus *P. brownii* of sect. *Onaepia* were obtained from GenBank. Using the cv. Oukan annotation as the reference, the annotations for the remaining 11 accessions were standardized.

Using PhyloSuite v1.2.2 (Zhang et al. 2020), sequences were extracted from the 12 chloroplast genomes for 77/78 protein-coding genes (Table S1). Multiple sequence alignments were then performed on each of the genes using MAFFT v7.475 (Zhang et al. 2020). Alignments for the individual genes were assembled into a single concatenated alignment with poorly aligned sections removed using Gblocks (Castresana 2000). Phylogenetic analyses were conducted using IQ-TREE v2.03 (Minh et al. 2020). A best-fitting nucleotide substitution model was first determined using the Akaike information criterion (AIC) using ModelFinder (Kalyaanamoorthy et al. 2017) as implemented in IQ-TREE (Minh et al. 2020). Finally, ML searches with 1000 bootstrap replicates were performed in IQ-TREE (Minh et al. 2020) using the best-fit model GTR + F + I + G4 and default run parameters. The phylogeny was visualized in ITOL (Letunic and Bork 2024).

## Results

**Figure 2.**
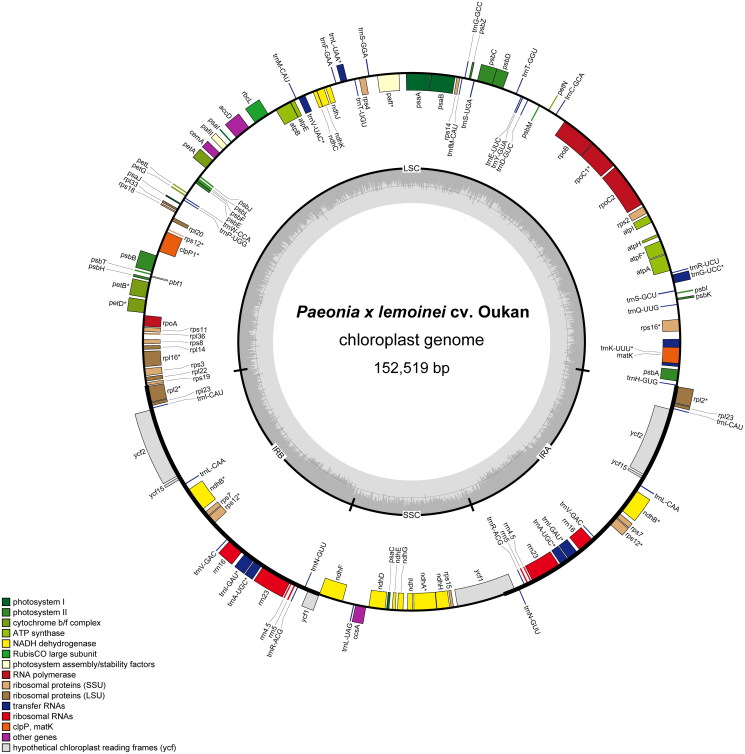
The chloroplast genome of *P. × lemoinei* cv. Oukan. Genes on the interior of the outer circle are transcribed in the forward direction; those on the exterior are transcribed in the reverse direction. The inner circle indicates the inverted repeat (IRa and IRb), the large single-copy (LSC) region and the small single-copy (SSC) region. The shaded ring on the interior of this circle indicates changes in %GC. Chloroplast genome drawn using OrganellarGenomeDraw ((Greiner et al. 2019).

The assembled chloroplast genome of *P. × lemoinei* cv. Oukan (Figure 2) was 152,519 bp in length and exhibited a typical quadripartite structure comprising a large single-copy (LSC) region (84, 213 bp), a small single-copy (SSC) region (17,026 bp), and a pair of inverted repeats (IRs) (25,640 bp each). Average read coverage across our assembled genome was 225.4 (Figure S1). The GC content was 38.4% overall with GC values for the LSC, SSC, and IRs being 36.74%, 32.78%, and 43.11%, respectively. Our annotation contains a total of 112 genes, comprising 78 protein coding, 30 tRNA, and four rRNA genes (Table S2). Among these, 18 fall within the IRs and are therefore duplicated. These include seven protein coding (i.e. *rpl2*, *rpl23*, *ycf2*, *ycf15*, *ndhB*, *rps7*, and *rps12*), seven tRNA (i.e. *trnI-CAU*, *trnL-CAA*, *trnV-GAC*, *trnI-GAU*, *trnA-UGC*, *trnR-ACG*, and *trnN-GUU*), and all four rRNA genes (i.e. *rrn4.5S*, *rrn23S*, *rrn5S*, and *rrn16S*). Eighteen genes contain introns. Of these them, nine protein-coding genes (i.e. *rps16*, *atpF*, *rpoC1*, *petB*, *petD*, *rpl16*, *rpl2*, *ndhB*, and *ndhA*) (Figure S2) and six tRNA genes (*trnK-UUU*, *trnG-UCC*, t*rnL-UAA*, *trnV-UAC*, *trnI-GAU*, and *trnA-UGC*) contain a single intron. Additionally, *clpP1*, *pafI* (Figure S2) and *rps12* contain two introns (Figure S3).

**Figure 3.**
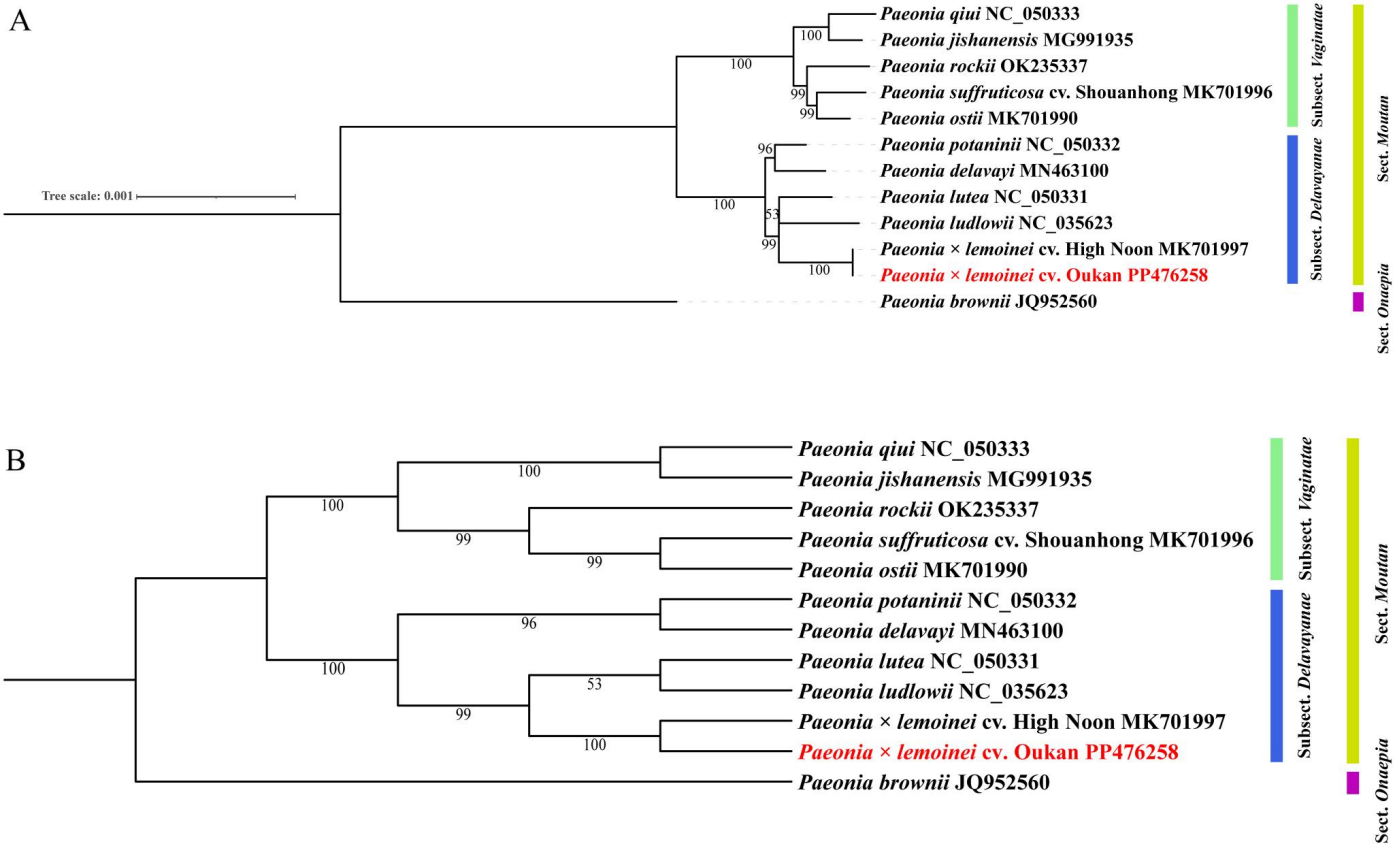
The ML topology based on the concatenated protein coding matrix presented as a phylogram (A) and cladogram (B). Maximum likelihood bootstrap values are associated with branches. In addition to cv. Oukan, the following sequences were used: *P. brownii* JQ952560 as outgroup (Chen et al. 2023), *P. delavayi* MN463100 (Chen et al. 2023), *P. jishanensis* MG991935 (Chen et al. 2023), *P. × lemoinei* cv. High Noon MK701997 (Guo et al. 2020), *P. ludlowii* NC 035623 (Guo et al. 2020), *P. lutea* NC 050331 (Guo et al. 2020), *P. ostii* MK701990 (Guo et al. 2020), *P. potaninii* NC 050332 (Li et al. 2018), *P. qiui* NC 050333, *P. rockii* OK235337, *P. suffruticosa* cv. Shouanhong MK701996 (Chen et al. 2023).

In our phylogenetic analysis, the included representatives of sect *Moutan* form two subclades, one containing members of subsect. *Vaginatae* and the other those of subsect. *Delavayanae*. Both subclades received bootstrap support (BS) of 100%. Within these two groups, all but one relationship was also strongly supported (i.e. BS 96–100%). The exception was the pairing of *P. lutea* and *P. ludlowii* that received a BS value of 53%. In our analysis, the triploids cv. Oukan (BS 100%) and cv. Shouanhong were sister to cv. High Noon (BS 100%) and *P. ostii* (BS 99%), respectively (Figure 3).

## Discussion and conclusions

The chloroplast genome of *P. × lemoinei* cv. Oukan is consistent, in terms of structure and sequence, with those previously reported for tree peonies. Noticeably, our cv. Oukan sequence shares 100% identity with that of its maternal parent, cv. High Noon; this is not unexpected given that chloroplasts are generally maternally inherited (Park et al. 2021) such as *Elaeis guineensis* (Mohd Talkah et al. 2024) and *Roscoea* (Hu et al. 2023).

In our phylogeny, members of subsect. *Vaginatae* and subsect. *Delavayanae* formed distinct subclades, consistent with phylogenetic analyses based on morphology (Hong 2010) and the previous phylogenies (Chen et al. 2018; Guo et al. 2020). In our analysis, the two triploid cultivars in sect. *Moutan*, cv. Oukan and cv. Shouanhong, fell within different subclades. Our accession of cv. Oukan was placed sister to its maternal parent, cv. High Noon within the subsect. *Delavayanae* clade whereas the naturally formed cv. Shouanhong (Li and Zhang 1982) was sister to *P. ostii* in the subsect. *Vaginatae* clade. Although in both cases, there is a sister relationship, the interpretation of these two situations is somewhat different. In the case of cv. Oukan, its placement is consistent with recent formation and cv. High Noon having been the maternal parent. In contrast, cv. Shouanhong is sister to but distinct from the included accession of *P. ostii*. Although this placement suggests *P. ostii* may represent the maternal parent, given that sampling of the genetically diverse *P. ostii* (Chen et al. 2018; Guo et al. 2020) is still limited, further work is needed to confirm this interpretation.

Our study supports the traditional morphology-based classification and adds support for evolutionary relationships within sect. *Moutan*. In terms of breeding guidance, our analyses suggest that lineages within both subsect. *Vaginatae* and subsect. *Delavayanae* are suitable for use as the maternal parents in breeding programs that aim to produce triploid tree peony cultivars.

## Supplementary Material

Supplementary materials.docx

## Data Availability

The genome sequence data that support the findings of this study are openly available in GenBank of NCBI at https://www.ncbi.nlm.nih.gov/ under the accession no. PP476258. The associated BioProject, SRA, and Bio-Sample numbers are PRJNA1126405, SRR29482263, and SAMN41941231, respectively.
