# Supplementary material for: The chloroplast genome of the allotriploid, Paeonia × lemoinei cv. Oukan, and its phylogenetic implications
Source: Mitochondrial DNA B Resour. 2025 Jun 23;10(7):615–9. doi: 10.1080/23802359.2025.2519229 (PMC12207776; doi:10.1080/23802359.2025.2519229)
Supplement: Supplementary materials.docx [file TMDN_A_2519229_SM5777.docx]

**Contents**

Figure S1. Sequence read coverage across the assembled chloroplast genome sequence of *P. × lemoinei* cv. Oukan.

Figure S2. Schematic maps showing the genome positions and orientation of the exons in the 13 cis-splicing protein coding genes in the *P. × lemoinei* cv. Oukan chloroplast genome.

Figure S3. Schematic map of the single trans-splicing gene, *rps12*, in the *P. × lemoinei* cv. Oukan chloroplast genome.

Table S1. List of protein coding genes included in concatenated matrix used in our phylogenetic analyses


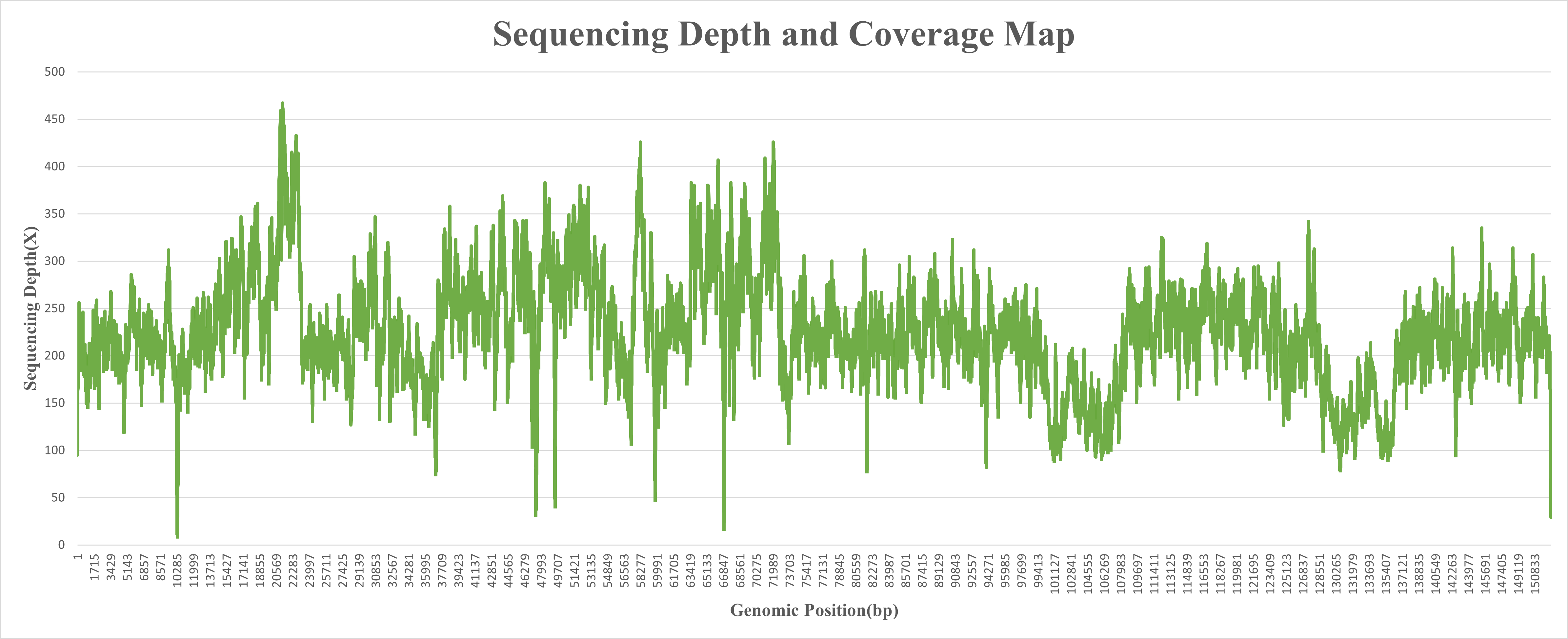


Figure S1. Sequence read coverage across the assembled chloroplast genome sequence of *P. × lemoinei* cv. Oukan

The minimum, average and maximum values of sequencing depth were 9, 225.4 and 467, respectively.


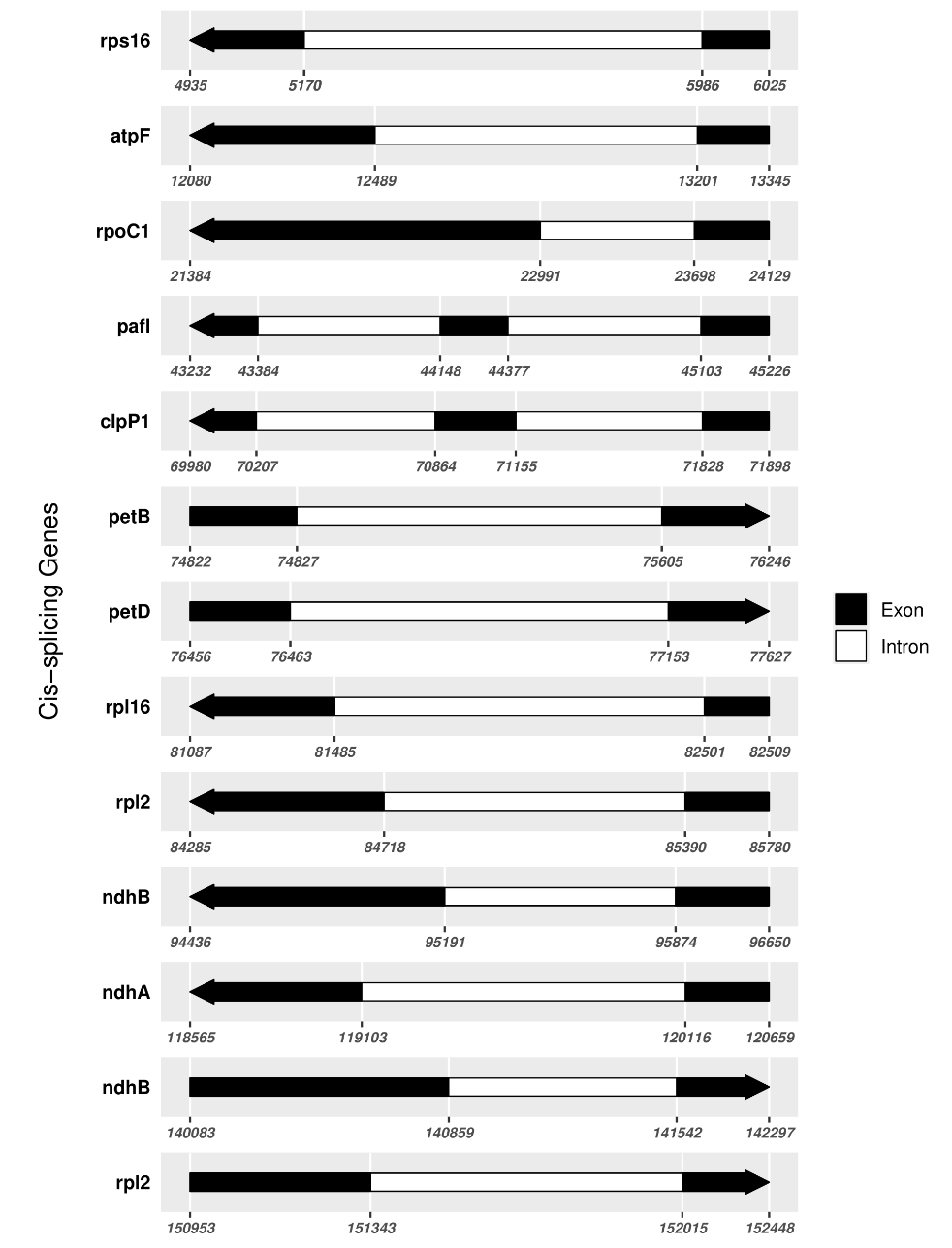


Figure S2. Schematic maps showing the genome positions and orientation of the exons in the 13 *cis*-splicing protein coding genes in the *P. × lemoinei* cv. Oukan chloroplast genome


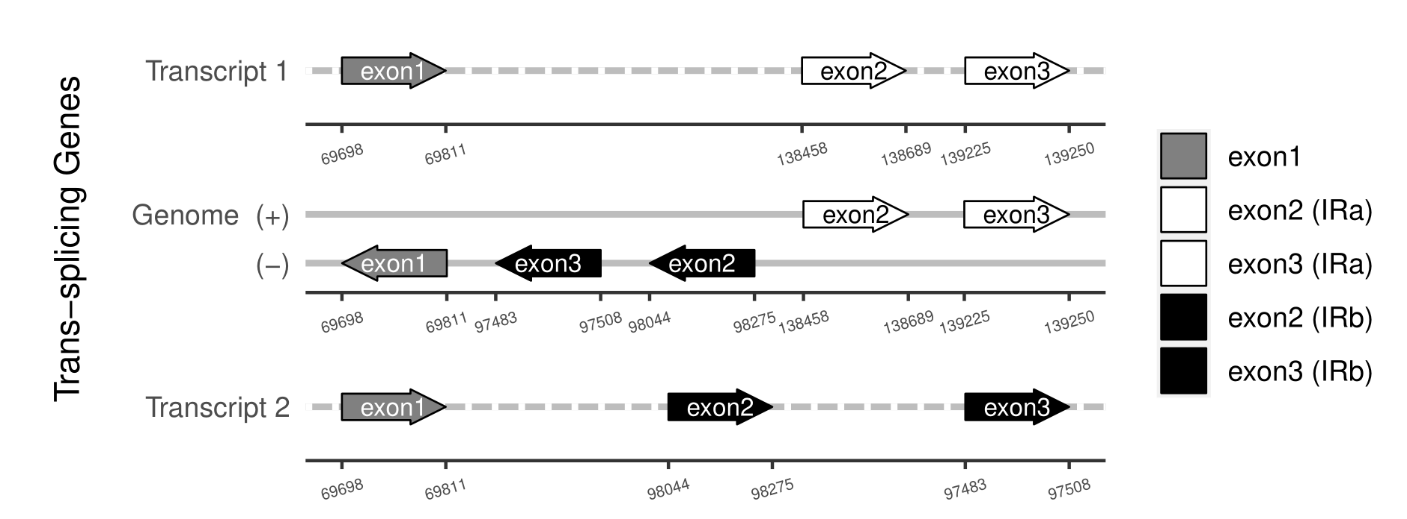


Figure S3. Schematic map of the single trans-splicing gene, *rps12*, in the *P. × lemoinei* cv. Oukan chloroplast genome

Table S1. List of protein coding genes included in concatenated matrix used in phylogenetic analyses

| Included protein-coding genes (77) | Not included protein-coding genes (1) |
| --- | --- |
| *ycf15, ycf2, ycf1, rps19, rps16, rps15, rps14, rps12, rps11, rps8, rps7, rps4, rps3, rps2, rpoC2, rpoC1, rpoB, rpoA, rpl36, rpl33, rpl23, rpl22, rpl20, rpl16, rpl14, rpl2, rbcL, psbZ, psbT, psbM, psbL, psbK, psbJ, psbI, psbH, psbF, psbE, psbD, psbC, psbB, psbA, psaJ, psaI, psaC, psaB, psaA, petN, petL, petG, petD, petB, petA, pbf1, paf*II*, paf*I*, ndhK, ndhJ, ndhI, ndhH, ndhG, ndhF, ndhE, ndhD, ndhC, ndhB, ndhA, matK, clpP1, cemA, ccsA, atpI, atpH, atpF, atpE, atpB, atpA, accD* | *rps18*^#^ |

^#^ The *rps18* was not detected in *P. brownie*. Consequently, it was excluded from the construction of the phylogenetic tree.Table S2. Annotation information of chloroplast genome of *P. × lemoinei* cv. Oukan

| Category | Gene group | Gene name |
| --- | --- | --- |
|  | Subunits of photosystem I | *psaA, psaB, psaC, psaI, psaJ* |
|  | Subunits of photosystem II | *psbA, psbB, psbC, psbD, psbE, psbF, psbH, psbI, psbJ, psbK, psbL, psbM, psbT, psbZ* |
| Photosynthesis | Subunits of NADH dehydrogenase | *ndhA*, ndhB*(2), ndhC, ndhD, ndhE, ndhF, ndhG, ndhH, ndhI, ndhJ, ndhK* |
|  | Subunits of cytochrome b/f complex | *petA, petB*, petD*, petG, petL, petN* |
|  | Subunits of ATP synthase | *atpA, atpB, atpE, atpF*, atpH, atpI* |
|  | Large subunit of rubisco | *rbcL* |
|  | Proteins of large ribosomal subunit | *rpl14, rpl16*, rpl2*(2), rpl20, rpl22, rpl23(2), rpl33, rpl36* |
|  | Proteins of small ribosomal subunit | *rps11, rps12**(2), rps14, rps15, rps16*, rps18, rps19, rps2, rps3, rps4, rps7(2), rps8* |
|  | Subunits of RNA polymerase | *rpoA, rpoB, rpoC1*, rpoC2* |
| Self-replication | Ribosomal RNAs | *rrn16(2), rrn23(2), rrn4.5(2), rrn5(2)* |
|  |  | *trnA-UGC*(2), trnC-GCA, trnD-GUC, trnE-UUC, trnF-GAA, trnG-GCC,* |
|  |  | *trnG-UCC*, trnH-GUG, trnI-CAU(2), trnI-GAU*(2), trnK-UUU*, trnL-CAA(2),* |
|  | Transfer RNAs | *trnL-UAA*, trnL-UAG, trnM-CAU, trnN-GUU(2), trnP-UGG, trnQ-UUG,* |
|  |  | *trnR-ACG(2), trnR-UCU, trnS-GCU, trnS-GGA, trnS-UGA, trnT-GGU,* |
|  |  | *trnT-UGU, trnV-GAC(2), trnV-UAC*, trnW-CCA, trnY-GUA, trnfM-CAU* |
|  | Maturase | *matK* |
|  | Protease | *clpP1*** |
|  | Envelope membrane protein | *cemA* |
| Other genes | Acetyl-CoA carboxylase | *accD* |
|  | c-type cytochrome synthesis gene | *ccsA* |
|  | other | *pafI**, pafII, pbf1* |
| Genes of unknown function | Conserved hypothetical chloroplast ORF | *ycf1, ycf15(2), ycf2(2)* |

Notes: Gene*: Gene with one introns; Gene**: Gene with two introns; Gene(2): Number of copies of multi-copy genes
